# Are Children Following High Trajectories of Disruptive Behaviors in Early Childhood More or Less Likely to Follow Concurrent High Trajectories of Internalizing Problems?

**DOI:** 10.3390/bs14070571

**Published:** 2024-07-05

**Authors:** Rene Carbonneau, Frank Vitaro, Mara Brendgen, Michel Boivin, Richard E. Tremblay

**Affiliations:** 1 Department of Pediatrics, University of Montreal, Montreal, QC H3T 1J7, Canada; 2 Centre de Recherche Azrieli du CHU Sainte-Justine, Montréal, QC H3T 1C5, Canada; 3 Research Unit on Children’s Psychosocial Maladjustment, University of Montreal, Montréal, QC H3T 1C5, Canada; 4 Department of Psychoeducation, University of Montreal, Montréal, QC H3C 3J7, Canada; 5 Department of Psychology, University of Quebec in Montreal, Montréal, QC H3C 3P8, Canada; 6 Department of Psychology, Université Laval, Québec City, QC G1V 0A6, Canada; 7 Department of Psychology, University of Montreal, Montréal, QC H3C 3J7, Canada

**Keywords:** co-occurrence, disruptive behaviors, internalizing problems, development, early childhood

## Abstract

The developmental association between disruptive behaviors (DBs: hyperactivity-impulsivity, non-compliance, physical aggression) and internalizing problems in early childhood is not well understood and has generated competing hypotheses and mixed results. Using a person-centered strategy, the present study aimed to examine concurrent trajectories of DBs and trajectories of internalizing problems from age 1.5 to 5 years in a population-representative sample (N = 2057; 50.7% boys). Six trajectories of DBs and three trajectories of internalizing problems, based on parent reports and obtained via latent growth modeling across five periods of assessment, were used as longitudinal indicators of each type of behaviors. Children following low or moderate trajectories served as the reference class. Compared to children in the reference class, those in trajectory classes characterized by high levels of co-occurring DBs (OR = 6.60) and, to a lesser extent, those in single high DB classes (OR = 2.78) were more likely to follow a high trajectory of internalizing problems simultaneously. These results support a multiple problem hypothesis regarding the association between DBs and internalizing problems, consistent with a developmental perspective that includes a general factor underpinning different psychopathologies. These findings highlight the importance of considering the co-occurrence between DBs and internalizing problems when studying either construct in children.

## 1. Introduction

Over the last two decades, longitudinal studies using a dual variable- and person-centered approach [1,2] have been useful in describing the development of disruptive behavior (DB) in childhood [3,4,5,6,7,8,9,10,11,12,13]. Overall, these studies support the idea that based on differences and similarities between children, a number of distinct developmental patterns of DB can be drawn across time (e.g., low, moderate or high stable, increasing, decreasing) and differentially linked with earlier risk factors or later outcomes. Most of these investigations examined single behaviors such as hyperactivity, opposition, or physical aggression, but a number of studies jointly examined the development of distinct DBs [3,10,11]. These latter studies generally showed a high level of co-occurrence of DBs, in addition to significant proportions of children displaying mainly one type of DB over time. Given the high risk of later psychosocial adjustment reported for children with concurrent DBs [3,14], these findings on the early development of such co-occurrence are important for early risk assessment and for screening children for prevention programs. However, another aspect of early developmental co-occurrence that may potentially differentiate trajectories of DBs is their association with internalizing problems [15] (which encompass inward-directed or overcontrolled behavior, such as mood disorders, anxiety or distress, and inhibition). Still, this association has received less attention in children’s early years. 

Recent advances in developmental psychopathology support a hierarchical structure that includes a common underlying factor of general psychopathology shared by all psychopathological conditions [16], while second-level factors influence externalizing and internalizing problems, respectively [17,18]. This model has been validated in behavioral genetic studies, showing a significant common genetic factor influencing both externalizing and internalizing problems [16,19]. Internalizing problems may thus develop concurrently with DBs from the first years of life, and such early co-occurrence therefore warrants investigation.

In comparison to studies focussing on DBs, a smaller number of investigations have examined longitudinally the co-occurrence between these latter problems and internalizing problems in children [20]. Moreover, results across studies are mixed. Indeed, some reports showed evidence of positive associations over time between externalizing and internalizing problems [21,22,23,24], suggesting cumulative conditions and supporting a *multiple problem hypothesis* [25]. Such results echo those of cross-sectional and longitudinal studies linking, for example, children’s anxiety and externalizing [26], oppositional-defiant behaviors and internalizing [27], or specific syndromes such as attention-deficit/hyperactivity and anxiety or depressive symptoms [28,29], as well as conduct or oppositional behaviors and anxiety or depressive symptoms [29,30,31]. Thus, DBs have been similarly associated with different forms of internalizing problems as well as global measures of internalization. Importantly, consistent with a multiple problem hypothesis, poorer long-term outcomes were reported for children exhibiting co-occurring internalizing and externalizing problems in comparison to their peers showing essentially one type of difficulty [23,32]. 

However, contrary to the aforementioned findings, other studies examining the developmental co-occurrence between DBs and internalizing problems in children have suggested that the presence of internalizing symptoms may have a dampening effect, aligning with a buffer hypothesis [25] on the development of DBs [33,34,35]. Such a buffering effect implies a negative association over time—opposite to the positive association involved in the multiple problem hypothesis—between DBs and internalizing problems. For instance, higher anxiety has been shown to moderate impulsive decision-making among individuals with high trait impulsivity [34]. In another study, children with externalizing traits and concurrent fearfulness/anxiousness at age 9 were less likely to develop aggressive behavior by age 12 than their peers with low fearfulness/anxiousness [35]. Consistent with the latter and supporting a buffer hypothesis, less-severe long-term outcomes were reported for children exhibiting concurrent internalizing and externalizing problems compared to their peers, who primarily exhibited externalizing problems [36,37].

The mixed support for the competing multiple problem or buffer hypotheses may be attributable, in part, to the cross-sectional nature of many reviewed studies and to the different ages of children at the time of assessment across studies. The developmental period considered may indeed affect the applicability of each explanation and potentially explain the mixed evidence. The mixed support for the competing hypotheses may also suggest that more than one developmental pathway involving the co-occurrence between both types of problems is possible. In this context, given the significant developmental heterogeneity described above concerning DBs, it may be particularly important to examine whether internalizing problems are similarly linked with different types or co-occurring longitudinal manifestations of DB. For instance, in a cross-sectional study of hyperactive boys aged 7–15, anxiety was associated with oppositional problems but not with symptoms of core conduct behavior, such as aggression [38]. Furthermore, a prior investigation based on the same sample as the one used in the present study showed that the association between preschool externalizing and internalizing problems varied depending on the type of externalizing profile among different DBs, the type of internalizing problems, and the specific preschool period (i.e., early, middle or late) [24]. Moreover, there are mixed results regarding sex differences in the association between externalizing and internalizing problems in early childhood, with some studies reporting a stronger association for boys [26,39], and others reporting no sex differences [27,40]. Given that several studies used at-risk or clinical samples, population-based studies are needed to clearly understand the association between developmental pathways of DBs and internalizing problems in the context of normative child development [41].

A comprehensive understanding of the co-occurrence between DBs and internalizing problems should start with the examination of individual patterns of their concurrent manifestations in children’s early years [15,20]. As early childhood is a developmental period marked by children’s accelerated maturation and changes in behaviors [42,43], a longitudinal appraisal of DBs and internalizing problems is necessary to avoid the caveats of cross-sectional assessments. To address this issue, a dual variable- and person-centered strategy allows us to describe the progression of the two types of problems using longitudinal data and to compare the behavior of the child to the behavior of his/her peers of the same age throughout early childhood [1,2]. The resulting longitudinal patterns thus provide a valid account of the development of a child with reference to the peer group for both types of problems. Such developmental patterns are a necessary prelude to comprehensive developmental models, including potentially common risk factors at the biological, individual, and sociofamilial level [42], which may distinguish DBs with and without concurrent internalizing problems [15], and which could eventually translate into early and personalized preventive interventions [43]. However, the potential concurrent development of DBs and internalizing problems in early childhood remains to be examined [26].

### The Present Study

**Figure 1:**
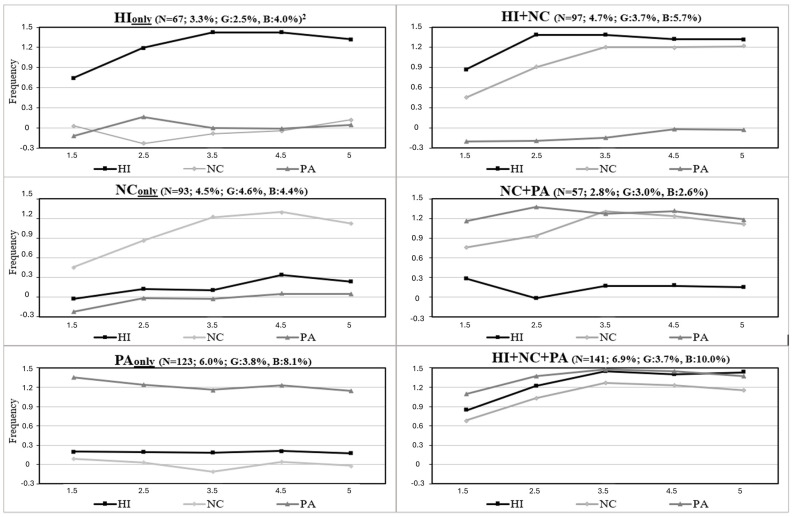
Observed standardized means^1^ for high single and co-occurrent trajectory classes of DBs from age 1.5 to 5 years Adapted from [3] Carbonneau et al., 2016 (http://creativecommons.org/licenses/by/4.0/, accessed on 28 June 2024). HI: hyperactivity–impulsivity; NC: non-compliance; PA: physical aggression. The co-occurrent trajectory class of HI+PA (i.e., without high NC) was not part of the observed joint trajectory classes. ^1^ Standardized scores. ^2^ % sample: girls; boys. From 1.5 to 5 years, the mean of the reference class (*n* = 1479; not shown above) ranged from −0.20 to −0.33 (average: −0.29) for HI, from −0.16 to −0.33 (average: −0.28) for NC, and from −0.23 to −0.28 (average: −0.31) for PA.

Aiming to help fill this gap, we examined the extent to which distinct developmental trajectories across different types of DBs are concurrently associated with the trajectories of internalizing problems over the early childhood years. To this end, we capitalized on young children’s longitudinal DBs trajectory classes identified in a previous study [3]. That study documented the joint longitudinal courses of mother-rated hyperactivity–impulsivity (HI), non-compliance (NC), and physical aggression (PA) using five assessments from ages 1.5 to 5 years in a representative birth cohort (Figure 1), as well as their association with teacher ratings of DBs in the first grade. The results showed that (1) for the three types of DBs, children followed either a low-, moderate-, or high-frequency trajectory throughout the early childhood years; (2) between 13% and 20% of children manifested higher levels of either HI, NC, or PA; (3) developmental co-occurrence between HI, NC, and PA was frequent, as 14.4% of the sample (boys: 18.3%; girls: 10.4%) followed the highest trajectory for more than one DB; and (4) early childhood longitudinal patterns of DBs were associated with teacher-rated indicators of DBs and school adjustment in the first grade. 

In the present study, we aimed to examine the association of these previously identified trajectories of DBs with the trajectories of internalizing problems over the same early childhood period in order to determine (1) whether the two types of trajectories are positively associated (i.e., in line with the multiple problems hypothesis), or, alternatively, whether they are negatively linked (i.e., in line with the buffer hypothesis); (2) whether such an association is more pronounced for concurrent or single manifestations of DB (i.e., HI, NC, or PA); and (3) whether these associations differ for males and females. 

The multiple problem hypothesis would be supported if trajectory classes reflecting high levels of DBs were linked with similar trajectories of internalizing problems rather than with classes with low or moderate levels of such problems. Moreover, children in trajectory classes reflecting high levels of concurrent DBs would be more likely to follow high trajectories of internalizing problems than their peers in high trajectory classes for a single DB. Conversely, the buffer hypothesis would be consistent with children in high DB trajectory classes being more likely to follow lower-level trajectories of internalizing problems than children in trajectory classes with a low or moderate frequency of DB. This hypothesis would also be consistent with children in high trajectory classes for a single DB showing higher levels of internalizing problems than their peers in classes of concurrent high DBs. 

Given that associations between DBs and internalizing problems have been reported for both global and specific measures of the latter (e.g., anxiety or depression symptoms), and given the high comorbidity consistently reported between anxiety and depression symptoms in early childhood [24,28,44,45], the present study focussed on a global indicator of internalizing problems in association with longitudinal patterns of DBs. Furthermore, given the absence of consensus noted earlier with regard to the multiple problem or buffer hypotheses, the lack of studies in young children on the association between patterns of single or concurrent high DBs with internalizing problems, and the mixed results regarding gender differences in prior studies, the present investigation remains exploratory with respect to these issues.

## 2. Materials and Methods

### 2.1. Participants

A population-based birth cohort of 2057 infants (92% white of European ancestry, 51.2% boys) representative of the children born (single births) in the province of Quebec, Canada, in 1997–1998 was used. Mothers giving birth after 24 weeks’ and not later than 42 weeks’ gestation who spoke English or French (Canada’s official languages) were eligible (French is the first language for 80% of the population in Quebec). A complete description of the cohort is available elsewhere [3]. Children and their families were first assessed 5 months after birth (T1) and annually thereafter. The annual assessments consisted of a face-to-face interview with the most knowledgeable person about the child (the mother in 99% of cases) over an average of one hour and forty-five minutes. Assessments at the ages of 1.5, 2.5, 3.5, 4.5, and 5 years (T2–T6; response rate of 97.7%, 95.4%, 95.1%, nd 86.0%, respectively) were used for trajectory analysis of DBs [3] and internalizing problems. Participants with valid trajectory data (*n* = 2057; 92.4%) were compared to those lost due to attrition (*n* = 169; 7.6%) with regard to demographic and socioeconomic family characteristics (i.e., mother’s and father’s age and family SES). A slightly higher SES of the remaining families was observed F(df = 1) = 17.72, *p* < 0.001, but the difference (Cohen’s *d* = 0.19) is considered to be extremely small (i.e., < 0.20) [46,47]. The characteristics of the participating families at the first assessment are presented in Supplementary Table S1. 

### 2.2. Measures

#### 2.2.1. Joint Trajectories of DBs

Trajectories of DBs were based on maternal ratings of hyperactivity–impulsivity (HI): cannot stand still; is agitated, fidgety, and/or impulsive; acts without thinking; difficulty waiting for his/her turn; difficulty remaining quiet; non-compliance (NC): is rebellious/defiant or refuses to comply with adults’ requests or rules; has no remorse after misconduct; does not change his/her behavior after being punished; and physical aggression (PA): fights; physically attacks others; hits, bites, or kicks. As recommended for scales with few items, internal consistency reliability was assessed by using confirmatory factor analysis to test the fit of a single factor model for each scale [48] from which a composite reliability coefficient (CR) [49] can be computed (>0.60 recommended) [50]. The average CR coefficient across assessment times was 0.81 (0.77–0.82) for HI, 0.78 (0.72–0.82) for NC, and 0.83 (0.81–0.85) for PA. A latent class growth mixture analysis [2,51] similar to that used in the present study (see Data analysis) generated the trajectory classes. 

Figure 1 shows the observed means of HI, NC, and PA for the six joint DBs trajectory classes that included a high-frequency trajectory for at least one DB. Overall, 34.8% of boys and 21.3% of girls were in joint DBs trajectory classes that included at least one high trajectory. Of note, a co-occurrent profile of HI+PA (i.e., without high NC) was not found. Among children following a high trajectory for a single-DB (HI, NC, or PA), the corresponding single high DB joint trajectory classes (i.e., high—HI_only_, NC_only_, or PA_only_) represented 25.3% to 36.2% of girls across DBs, and 19.4% to 39.1% of boys. Thus, for both genders, trajectory classes of co-occurrent DBs (i.e., including a high trajectory for two or more DBs: High—HI+NC, NC+PA, HI+NC+PA) represented the majority of children following a High trajectory within each DB. 

#### 2.2.2. Internalizing Problems

Measures of internalizing problems were based on maternal ratings of items originating from the Preschool Behavior Questionnaire [52] and the Child Behavior Checklist 2/3 [53] and were previously used in large population studies [24,54,55,56]. The items describe manifestations of anxiety, separation anxiety, and emotional or depressive problems; if the child is too fearful or anxious; is worried; cries a lot; is nervous or very tense; clings to adults or too dependent; does not want to sleep alone; reacts very badly away from his parents; seems unhappy or sad/not as happy as other children; has trouble having fun. Measures were collected at the same five assessment times as the DBs data from age 1.5 to 5 years. All items were rated on a 3-point scale: 0/*never*; 1/*sometimes*; and 2/*often*. Confirmatory factor analysis was used to assess the construct validity of a global measure of internalizing problems. The average fit indicators over the 5 assessment times were 0.056 (range: 0.05–0.06) for RMSEA, 0.88 (0.86–0.89) for CFI, 0.85 (0.83–0.86) for CLI, and 0.038 (0.03–0.04) for SRMR, indicating an overall acceptable fit. Average loading from factor analysis was 0.520 (range 0.500–0.545) across the 5 assessment times. Thus, items were summed into a global internalizing score at each assessment time. The average CR coefficient of internal consistency reliability across assessment times was 0.78 (range: 0.73–0.81). The average correlation between successive assessment times for the internalizing scale was 0.50 (range: 0.45–0.55, *p* < 0.001).

### 2.3. Data Analysis

Following previous studies investigating the link between individual characteristics and trajectories of DBs [4,5,11,24], we compared children on a high vs. a low or moderate trajectory of DBs. Thus, children in the high-DB trajectory classes (Figure 1)—either for a single DB (i.e., high—HI_only_, NC_only_, or PA_only_) or for co-occurrent DBs (high—HI+NC, NC+PA, HI+NC+PA)—were compared to a reference class (*n* = 1479) that aggregated their peers who followed low or moderate trajectories for all DBs across the preschool years. Of note, the moderate trajectory was the modal category, followed by the Low category, for all three DBs [3]. Class members’ posterior assignment probabilities to trajectory classes were used as sampling weights in the analysis to account for uncertainty in the assignment of individuals to trajectory classes [2,57]. 

On the basis of the data collected at the five assessment times for all children in the sample, a similar method to that used to estimate trajectory classes of DBs was performed for internalizing problems. To this end, latent class growth mixture modeling (LCGMM) in Mplus/version−8.6 [51] was used. In a first step, a latent growth curve model (LGCM) of children’s level and growth in internalizing problems across time was tested. 

Interindividual variability in internalizing problems is estimated as the variance of the latent intercept (i.e., the initial level at time 1) and of the latent slope (i.e., change in behavior across assessment times). To assess this first model, where all individuals are essentially considered part of the same “class”, the significance levels of the mean and variance of the intercept, linear slope, and quadratic slope, as well as their covariance, were examined. The variances suggested significant heterogeneity within the sample with regard to children’s level (intercept) and the rate of change across time (slope) of the internalizing problems (see Section 3). Therefore, LCGMM (an extension of LGCM) was used to test whether a better fit would be obtained with the estimation of two or more latent classes of children who have different intercept or slopes of internalizing problems. To this end, subsequent models were tested sequentially, starting with comparing the fit of a 2-class model (k) to the initial 1-class model (k−1). If the 2-class model showed an improvement in fit, suggesting that aggregating individuals in 2 classes better fit the data than a single-class model, a 3-class model was tested, and so on. This was repeated until the k class model did not show a further improvement in fit, indicating that the prior model (k−1) would be the best-fitting model.

The LCGMM procedure was conducted in accordance with the recommended guidelines [58], testing different trajectory models starting with latent class growth analysis (LCGA, which assumes no intra-class variance within the distinct latent classes) to determine whether constraints can be applied to simplify the number of parameters. Model-fit was determined by (1) information criteria statistics: Akaike information criterion (AIC), Bayesian information criterion (BIC), and consistent Akaike information criterion (CAIC); (2) likelihood ratio test (LRT) and adjusted-LRT to identify the optimal number of classes; (3) two indicators of classification: entropy, which indexes class separation, and the average probabilities of assignment (APA) of individuals in trajectory classes (satisfactory when ≥0.70) [2]; and (4) relative class-size above 5% of the sample to avoid selecting an over extracted and potentially unstable class solution, most likely to be sample-specific with poor replicability [59].

In the last step, to examine the association between trajectory classes of DBs and trajectories of internalizing problems, multivariate analyses based on a generalized linear models (GLMs) platform were used. GLMs can be used alternately with normal or non-normal data with skewed distributions [60] and provide information criteria (IC) that include penalties for the number of model parameters, which leads to adjusted estimates. A model including sex and its potential interaction with DB trajectory classes was fit first to examine each term’s contribution within the multivariate context and to obtain IC representing initial fit statistics. For all IC, the smallest value indicates the best model. Models with IC differences of less than 2 provide little evidence for favoring one over the other, whereas a difference of 10 or more is considered strong evidence [61]. Based on estimates of predictor coefficients and significance levels and variations in fit statistics, the model was sequentially reduced to the most parsimonious combination of predictors (i.e., trajectory classes of DBs, with or without sex and their interaction) in association with trajectories of internalizing problems [62].

## 3. Results

### 3.1. Longitudinal Growth Curve Model and Trajectory Classes of Internalizing 

The best-fitting growth curve model showed significant intercept (2.175, *p* < 0.001), linear (0.996, *p* < 0.001), and quadratic (−0.091, *p* < 0.001) slopes. This indicated that all three growth parameters were part of the model describing the sample’s development of internalizing behaviors over time. The variances of the intercept (3.79, *p* < 0.001) and of the linear (1.15, *p* < 0.001) and quadratic (0.037, *p* = 0.018) slopes were also statistically significant. In other words, there was significant heterogeneity among children in the initial level and course of internalizing problems. This finding justified extending the analyses to test whether a better fit would be obtained with the estimation of two or more latent classes of children with distinct levels and rates of internalizing problems growth.

Using LCGMM, one- through four-class models were tested, along with alternative link functions based on normal and censored normal distributions, given the positive skewness of the internalizing problems data. The best solution was a censored normal link-based three-class quadratic model, with a fixed variance–covariance matrix structure within classes (LCGA) and a fixed variance–covariance matrix structure across classes (Table 1). The fit of this model was better than those of models based on a normal distribution, free variance–covariance matrix structure within classes (growth mixture model) and linear parameterization. Despite the improvement in IC values and significant LRT results observed in the four-class model, its classification indicators were below the tolerance level for entropy (E = 0.66 < 0.70) and lower for APA (mean = 0.78, range: 0.72 – 0.84), and one of the classes represented only 2.2% (*n* = 46) of the sample. The comparison with the three-class solution (E = 0.72; mean APA = 0.85, range: 0.83 – 0.89) pointed to the latter as the best model for subsequent analyses.

**Figure 2:**
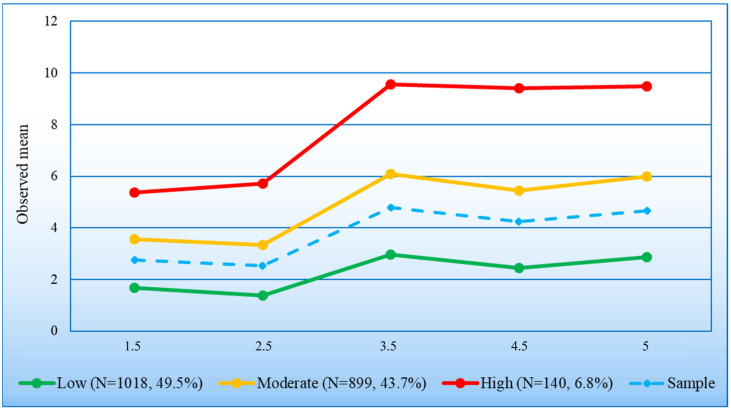
Trajectory classes^1^ of internalizing problems from age 1.5 to 5 years (*n* = 2057). ^1^: % girls (G); boys (B). Low trajectory: G: 47.9; B: 52.1. Moderate trajectory: G: 51.2; B: 48.8. High trajectory: G: 47.1; B: 52.9. Sample: G: 49.3; B: 50.7.

The three trajectory classes of the selected model are shown in Figure 2. Overall, children in all three classes showed a fairly similar trend over time, although the curves become increasingly pronounced (or less flattened) from the lower trajectory class to the higher trajectory class. Specifically, a slight decline between 1.5 and 2.5 years for the two lower classes and a slight increase for the higher class were followed by a significant increase for all three classes up to age 3.5 years and then by relative stability until the age of 5 years. Trajectory classes clearly differed in their level of internalizing problems, particularly from age 2.5 years onwards. The low (49.5% sample; modal trajectory class) and moderate (43.7%) classes each had large proportions of participants with respective average scores below (T1–T5 Average: −0.62 SD) or somewhat above (0.48 SD) the sample mean, representing 93.2% of preschoolers. The third, high trajectory class included 6.8% of children, which were consistently rated well above (1.61 SD) the sample mean. Expressed in proportion of sample SD, the average difference between the low and moderate classes across times of assessment was 1.05 SD (range: 0.84–1.17), 1.18 (range: 0.76–1.48) between the moderate and high classes, and 2.23 (range: 1.60–2.63) between the low and high classes. There were no significant differences in the proportion of boys and girls across trajectory classes (*X*^2^(2) = 2.40, *p* = 0.30).

### 3.2. Associations between Trajectory Classes of DBs and Trajectory Classes of Internalizing Problems

Table 2 shows the proportion of children in the different trajectory classes of high DBs and in the DB reference trajectory class (i.e., children who followed a low or moderate trajectory for all DBs) who simultaneously followed a low, moderate, or high trajectory of internalizing problems from age 1.5 to 5 years. While the moderate internalizing class was the modal class for children of all high DB trajectory classes, a majority (56.0%) of children of the DB reference class were in the low internalizing problems class. Furthermore, at 3.7%, the proportion of children of the DB reference class simultaneously in the higher class of internalizing problems was lower than that observed for children of the high DB classes (range: 7.3–22.7%). Among the latter, children of the high—HI+NC and HI+NC+PA trajectory classes stand out, with 20.6% and 22.7%, respectively, also following a high trajectory of internalizing problems over the early childhood years compared to 10.4%, on average (range: 7.3–12.9%), for their peers in the other high DB trajectory classes.

To examine further the associations between trajectory classes of DBs and classes of internalizing problems, the former were used as the independent variable and the latter as the dependent variable in GLM analysis using sex as a covariate, along with its potential interaction with DB trajectory classes. The model including solely DB classes as a predictor of internalizing trajectory class showed a better fit (∆AIC = 1.886; ∆BIC = −37.517; ∆CAIC = −44.517) than the models that also included sex and/or its potential interaction with DB trajectory classes (see Table S2). This suggests that the associations between trajectory classes of DB and classes of internalizing problems did not vary between boys and girls. The odds ratios (OR) derived from the selected model are shown in Table 2. Children in all high DB trajectory classes showed higher odds of simultaneously following a moderate (rather than a low) trajectory of internalizing problems than their peers in the DB reference class (i.e., children who followed a low or moderate trajectory for all DBs), with average ORs of 1.97 for high single-DB classes and 2.66 for high concurrent DBs classes. They also showed higher odds of simultaneously following a high (rather than a low) trajectory of internalizing problems, with average ORs of 4.04 for high single-DB classes and 10.52 for high concurrent DBs classes. Children in trajectory classes of HI+NC (OR: 14.6) and HI+NC+PA (OR: 11.6) showed the highest odds. Finally, the odds of simultaneously following a high rather than a low or moderate trajectory of internalizing problems were, on average, 2.86 for high single-DB classes and 6.10 for high concurrent DBs classes compared to their peers in the DB reference class. Children in trajectory classes of HI+NC (OR: 6.85) and HI+NC+PA (OR: 7.75) showed the highest odds.

**Figure 3:**
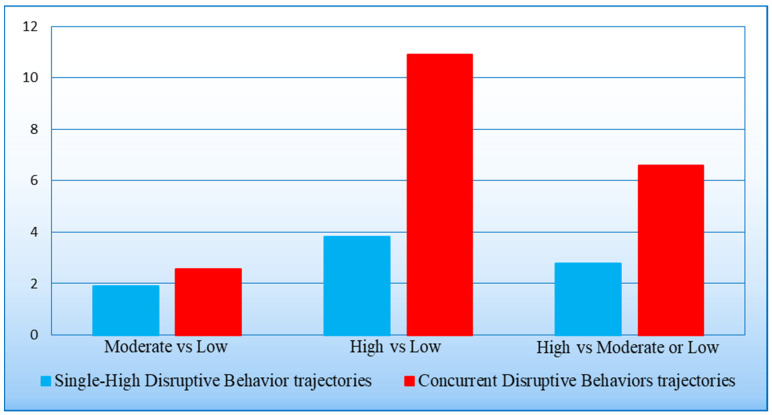
Estimated odds of simultaneously following a moderate or a high trajectory of internalizing problems from age 1.5 to 5 years in comparison to the reference class.

When grouping children into a concurrent high DB class and a single-high DB class (Figure 3), the ORs were 2.56 and 1.90, respectively (both at *p* < 0.001), simultaneously following a moderate (rather than a low) trajectory of internalizing problems, compared to their peers of the DB reference class—a non-significant (*p* = 0.391) difference in odds. Comparing the odds of the two aggregated classes to simultaneously following a high (rather than a low) trajectory of internalizing problems, the ORs were 10.90 and 3.83, respectively (*p* < 0.001)—a significant (*p* = 0.002) difference in odds. Finally, the ORs were 6.60 and 2.78, respectively (*p* < 0.001), simultaneously following a high trajectory of internalizing problems (rather than a low or moderate trajectory), compared to their peers of the DB reference class—a significant (*p* = 0.007) difference in odds.

## 4. Discussion

The aim of the present study was to investigate whether distinct developmental trajectories of DBs were concurrently and differentially associated with trajectories of internalizing problems in children from age 1.5 to 5 years. Overall, the findings are consistent with a multiple problem hypothesis, which posits that internalizing symptoms are positively associated with DBs [25]. Thus, the results with our population-representative sample did not provide evidence that internalizing problems may have a dampening effect (i.e., the buffer hypothesis) on the development of DBs. Specifically, children in all trajectory classes of high DBs were more likely to simultaneously follow a higher trajectory of internalizing problems throughout the early childhood years than their peers of the DB reference class following low or moderate DB trajectories. This finding was more marked for children in trajectory classes of high—HI+NC and HI+NC+PA and, on average, for those in DB trajectory classes of any high co-occurring DBs than for their peers in classes showing a single high-DB.

Thus, with regard to a differential association of internalizing problems with distinct types of DB, the results suggest that the two DB trajectory classes more likely to also follow the highest trajectory of internalizing problems were those comprising high HI+NC (i.e., HI+NC and HI+NC+PA). In comparison, the other class with co-occurring DBs, high NC+PA, showed odds of following a high internalizing trajectory closer to the range observe for single-high DB classes. These results suggest that a high level of HI combined with a high level of NC in young children is a pattern of DB likely to be associated with higher internalizing problems. This finding is consistent with prior reports suggesting that children struggling with negative emotionality [25,63,64], irritability [65,66], emotion regulation [67,68], or executive functions and effortful control [69,70] are more likely to show both externalizing and internalizing problems. Indeed, previous research found stronger associations of such endophenotypes representing heritable internal liabilities [71] with symptoms of either hyperactivity–inattention or opposition than with symptoms of aggression [38,72]. Consistent associations with internalizing symptoms were also observed [25,65,66]. Thus, among children with DB, those exhibiting high HI and NC may be more likely to struggle with both behavioral and emotional self-control. However, more research is needed in order to understand to what extent the specific endophenotypes involved in the mechanisms that underlie this early emotional–behavioral developmental pattern involve liabilities for experiencing negative emotions, for expressing discontent and anger in response to frustrations, or for difficulties managing emotions or behaviors.

Of note, although the present study was not designed to formally test this issue, the concurrent higher levels of internalizing problems observed in young children with high DBs levels from the age of 1.5 years provide very limited support for the hypothesis that one type of problems may precede the other at this early age period [25]. Rather, the results suggest that this co-occurrence is rooted earlier in children’s development, which likely involves intergenerational transmission through either or both genetic [16,19] and pre- to early-post-natal environmental risk factors, consistent with a developmental biopsychosocial perspective [42,43]. This early dual pathway is also consistent with the existence of a general, highly heritable underlying factor common to different forms of psychopathology, in addition to specific-second level factors influencing externalizing and internalizing problems [16,17,18]. Scholars have suggested that endophenotypes such as negative emotionality, irritability, emotion regulation, or effortful control discussed above in relation to DBs and internalizing problems [63,66,67,69,70] may be intermediates of this general factor’s influence on concurrent DBs and internalizing problems [18,19].

At the same time, on average, a third (33.2%) of children in high DBs trajectory classes (i.e., 37.5% in single high DB classes and 28.8% in high concurrent DBs classes, representing 9.3% of the sample) concurrently followed the modal low trajectory of internalizing problems, which includes nearly half (49.5%) of the sample. It is thus possible that some of these children develop later internalizing symptoms as a consequence of early life-course experiences tainted by their DBs problems, as suggested in previous reports [25]. Conversely, however, 3.7% of children in the reference class (following either a low or moderate trajectory for all DBs), representing 2.6% of the sample, concurrently followed the high trajectory of internalizing problems (6.8% sample). Thus, although the present study did not specifically test this, the results suggest that the likelihood of a significant proportion of children with high internalizing problems and without high DB in early childhood subsequently developing DBs is much less strong than the reverse, i.e., children with high DBs and without high internalizing in early childhood developing future internalizing problems. Future studies based on the long-term follow-up of young children, as well as studies across different developmental periods, are needed to clarify this issue.

Given the higher risk of later social maladjustment for youth showing concurrent DBs and internalizing problems [23,32], the present findings suggest that children showing a higher level of both DBs and internalizing problems from the second year of life onward are an important group to screen for a more comprehensive assessment and potentially to target for preventive intervention aimed at reducing early risk factors [73,74]. This seems to apply equally to both boys and girls, as no significant sex difference was found regarding the association between the DBs trajectories and trajectories of internalizing problems. Similar results were observed in preschool and elementary school children in prior reports [23,24,27]. However, as sex differences in internalizing problems are known to emerge later [75], it is possible that the association between concurrent patterns of DBs and internalizing problems differs over time for boys and girls, and this should be the focus of further research. In a related vein, future research should examine the extent to which different levels of concurrent DBs and internalizing problems in young children may be differentially associated with later outcomes. Such an investigation is important, as there is mixed evidence regarding the potential additive, synergetic, or buffering influence of high concurrent DBs and internalizing problems, for instance, with regard to adolescent substance use [36,37,76]. Such long-term follow-up may also help determine whether the early co-occurrence between the two types of problems represents an early manifestation of a specific developmental pathway toward later psychopathology or not.

### Strengths and Limitations

The large population cohort and the longitudinal patterns based on multiple assessments to represent DBs and internalizing problems are important strengths of the present study. However, several limitations should be noted. First, relying essentially on parental reports to assess children’s DBs and internalizing problems may implicate participants’ self-presentation concerns, recall bias, or halo effect. However, as in other similar large-scale studies, there is no easily available alternative when assessing large population-based samples at this early age, and parental reports remain the most widely used method for surveys in the general population [4,56]. Of note, parental—often maternal—reports of young children’s behavioral and emotional well-being from the first year of life throughout early childhood have been consistently shown to be valid assessments, even at such an early age [63,77,78]. 

Second, despite the advantage provided by the longitudinal patterns, the method used requires the examination of the same behaviors across different time points, and there are obvious constraints regarding which behaviors could be reasonably and meaningfully assessed in children from age 1.5 to 5 years. However, other non-measured behaviors may have captured additional developmentally based distinctions among children. Third, as this was an exploratory study of the association between DBs trajectories and trajectories of internalizing problems, a global measure of the latter was used. As noted, this is consistent with previous reports of the association of DB with both global and discrete measures of internalizing problems, as well as the high comorbidity reported between the latter [24,29,44,45]. Thus, the results should be interpreted in this context and the potential specificity of such an association for one type of internalizing problem or another will require further investigation. Fourth, the results of LCGMM have been criticized for not being easily replicated across studies and samples [79]. The lack of methodological details provided in many studies and the disparity in samples, criteria, and indicators used likely account for this difficulty in replication [79]. Hence, we followed the recommendations for conducting analyses and reporting in latent trajectory studies, such as reporting on preliminary steps of analysis and on alternative models tested and using a range of indices and criteria when enumerating classes [58]. Fifth, this study was based on a North American population sample of predominantly white and mostly French-speaking families of European ancestry. Replications are needed to examine the generalizability of the results to different populations and children of diverse cultural and social backgrounds.

The results of the present study, which jointly examined the longitudinal patterns of DBs and the concurrent patterns of internalizing problems throughout early childhood, suggest that these two types of problems in children develop concurrently from the age of 1.5 onwards. Thus, the findings support a multiple problem hypothesis regarding the early association between DBs and internalizing problems [25]. They also highlight the importance of considering the developmental co-occurrence between these two types of problems when studying either construct in children [80]. Future studies across diverse populations, developmental stages, and subgroups of individuals may help elucidate the discrepancies between reports regarding the competing “multiple problem” or “buffer” hypotheses [25,27], explaining the association between disruptive behaviors and internalizing problems in children.

## Figures and Tables

**Table 1:** Results from LCGMM analysis of internalizing problems from age 1.5 to 5 years.

Model	LCGA				LCGMM
Distribution	Normal		Censored Normal		Censored Normal
Fit Indicators	Three classes	Four classes	Three classes	Four classes	3-classes
AIC	44,193.09	43,993.98	43,535.35	43,415.23	43,547.35
BIC	44,283.15	44,106.56	43,625.41	43,527.81	43,671.19
SSA-BIC	44,232.32	44,043.02	43,574.58	43,464.26	43,601.29
VLMR-LRT (*p*)	0.003	0.003	0.034	0.003	0.035
LMR-ALRT (*p*)	0.004	0.003	0.037	0.003	0.036
Entropy	0.74	0.74	0.72	0.66	0.70

**Table 2:** Proportion (%) of children in sample, high DB, or DB reference trajectory classes following a low, moderate, or high trajectory of internalizing problems from age 1.5 to 5 years, and estimated odds of following a moderate or high internalizing trajectory in comparison to children of the DB reference class.

DB Trajectory Classes (*n*)	Trajectory Classes of Internalizing Problems			Moderate vs. Low			High vs. Low			High vs. Low or Moderate		
	Low (1019)	Moderate (899)	High (139)	Sig.	OR	OR 95% CI	Sig.	OR	OR 95% CI	Sig.	OR	OR 95% CI
DB reference class ^1^ (1479)	56.0	40.4	3.7									
HI_only_ (67)	34.3	56.7	9.0	0.002	2.29	1.35–3.89	0.004	4.00	1.56–10.24	0.034	2.60	1.08–6.27
NC_only_ (93)	36.6	50.5	12.9	0.005	1.92	1.22–3.02	<0.001	5.41	2.65–11.05	<0.001	3.91	2.01–7.60
PA_only_ (123)	41.5	51.2	7.3	0.006	1.71	1.17–2.52	0.010	2.71	1.27–5.79	0.049	2.08	1.03–4.33
HI+NC (97)	21.6	57.7	20.6	<0.001	3.70	2.22–6.17	<0.001	14.60	7.46–28.58	<0.001	6.85	3.91–12.02
NC+PA (57)	35.1	52.6	12.3	0.013	2.08	1.17–3.70	<0.001	5.37	2.17–13.25	0.002	3.69	1.60–8.53
HI+NC+PA (141)	29.8	47.5	22.7	<0.001	2.21	1.48–3.30	<0.001	11.6	6.84–19.97	<0.001	7.75	4.80–12.50
Sample (2057)	49.5	43.7	6.8									

Odds ratio (OR): Estimated odds of following a moderate or high trajectory of internalizing problems versus a low trajectory, and a high versus a low or moderate trajectory. ^1^: The DB reference class includes children who followed a low or moderate trajectory for all DBs.

## Data Availability

Data used in the present study are owned by Gouvernement du Québec and are under the responsibility of Institut de la statistique du Québec. Data cannot be shared publicly and are protected by the ACCESS TO INFORMATION ACT (Québec), as indicated in the consent form signed by the participants and approved by the Ethics Committee of Santé Québec Division. Data are available from the Centre d’accès aux données de recherche de l’Institut de la Statistique du Québec (CADRISQ) for researchers who meet the criteria for access to confidential data. Please contact Nancy Illick: nancy.illick@stat.gouv.qc.ca. See also: http://www.jesuisjeserai.stat.gouv.qc.ca/informations_chercheurs/acces_an.html.

## Supplementary Materials

The following supporting information can be downloaded at https://www.mdpi.com/article/10.3390/bs14070571/s1, Table S1: Socioeconomic characteristics of the families at the first assessment time; Table S2: Results of GLM model testing for the association between trajectory classes of DBs and trajectory classes of internalizing problems.
