# Supplementary material for: Are Children Following High Trajectories of Disruptive Behaviors in Early Childhood More or Less Likely to Follow Concurrent High Trajectories of Internalizing Problems?
Source: Behav Sci (Basel). 2024 Jul 5;14(7):571. doi: 10.3390/bs14070571 (PMC11274135; doi:10.3390/bs14070571)
Supplement: Supplementary file 1 [file behavsci-14-00571-s001.zip › behavsci-3050871-supplementary.pdf]

Supplemental materials

Are children following high trajectories of disruptive behaviors in early childhood more or less likely to follow concurrent high trajectories of internalizing problems?

Table S1

Socioeconomic characteristics of the families at the first assessment time[1] (N=2057)

Families Socioeconomic Characteristics	%
Household Income	
- < CAD 30,000	32.8
- CAD 30,000 - CAD 59,000	39.6
- CAD 60,000 and +	27.6
Family status	
- Intact/two-parent family	80.0
- Non-Intact family	20.0
Number of children in household	
- No brother or sister	41.7
- 1 brother or sister	40.0
- 2 or more	18.3
Education: Mother	
- No high school diploma	17.9
- High school or Technical school diploma	22.1
- Post-secondary education	60.0
Education: Father	
- No high school diploma	17.6
- High school or Technical school diploma	24.2
- Post-secondary education	58.2
Age Group of Mother	
- < 20 years	3.3
- 20-39 years	94.3
- 40 years and +	2.4
Age Group of Father	
- < 20 years	0.5
- 20-39 years	91.0
- 40 years and +	8.5
Race	
- Caucasian	92.1
- Other	7.9

[1]: Adapted from Carbonneau et al., 2016 (http://creativecommons.org/licenses/by/4.0/).

Table S2

Results of GLM model testing for the association between trajectory classes of DBs and trajectory classes of internalizing problems

Model			
Full model	X^2	df	sig.
Trajectory classes	125.316	6	<.001
Sex	2.361	1	.124
Interaction	8.220	6	.222
Model fit (LR)	154.066	13	<.001
AIC		159.905	
BIC		244.340	
CAIC		259.340	
Final model	X^2	df	sig.
Trajectory classes	131.963	6	<.001
Model fit (LR)	138.180	6	<.001
AIC (ΔAIC)		161.791 (1.886)	
BIC (Δ BIC)		206.823 (-37.517)	
CAIC (Δ CAIC)		214.823 (-44.517)	

Likelihood ratio (LR); Akaike information vriterion (AIC); Bayesian information criterion (BIC); consistent Akaike information criterion (CAIC). Δ AIC, Δ BIC and Δ CAIC are derived from comparison with the full model.
